# Transition of Serotype 35B Pneumococci From Commensal to Prevalent Virulent Strain in Children

**DOI:** 10.3389/fcimb.2021.744742

**Published:** 2021-10-26

**Authors:** Naoko Fuji, Michael Pichichero, Rachel L. Ehrlich, Joshua Chang Mell, Garth D. Ehrlich, Ravinder Kaur

**Affiliations:** ^1^ Center for Infectious Diseases and Immunology, Rochester General Hospital Research Institute, Rochester, NY, United States; ^2^ Department of Microbiology and Immunology, Drexel University College of Medicine, and Center for Genomic Sciences, Institute of Molecular Medicine and Infectious Disease, Philadelphia, PA, United States; ^3^ Department of Otolaryngology–Head and Neck Surgery, Drexel University College of Medicine, Philadelphia, PA, United States

**Keywords:** *S. pneumoniae*, serotype 35B, antibiotic resistance, β-lactams, fluoroquinolones, whole genome sequencing, comparative genomics, acute otitis media

## Abstract

In our community-based prospective cohort study in young children, we observed a significant increase in pneumococcal serotype 35B nasopharyngeal (NP) commensal colonization during the 2011–2014 timeframe, but these strains were not associated with disease. Beginning in 2015 and continuing through to the present, the serotype 35B virulence changed, and it became the dominant bacteria isolated and associated with pneumococcal acute otitis-media (AOM) in our cohort. We performed comparative analyses of 250 35B isolates obtained from 140 children collected between 2006 and 2019. Changes in prevalence, clonal-complex composition, and antibiotic resistance were analyzed. Seventy-two (29%) of 35B isolates underwent whole-genome sequencing to investigate genomic changes associated with the shift in virulence that resulted in increased rates of 35B-associated AOM disease. 35B strains that were commensals and AOM disease-causing were mainly associated with sequence type (ST) 558. Antibiotic concentrations of β-lactams and ofloxacin necessary to inhibit growth of 35B strains rose significantly (2006–2019) (p<0.005). However, only isolates from the 35B/ST558 showed significant increases in MIC_50_ of penicillin and ofloxacin between the years 2006–2014 and 2015–2019 (p=0.007 and p<0.0001). One hundred thirty-eight SNPs located in 34 different genes were significantly associated with post-2015 strains. SNPs were found in *nrdG* (metal binding, 10%); *metP* and *metN* (ABC transporter, 9%); *corA* (Mg^2+^ transporter, 6%); *priA* (DNA replication, 5%); and on the enzymic gene *ldcB* (LD-carboxypeptidase, 3%). Pneumococcal serotype 35B strains was a common NP commensal during 2010–2014. In 2015, a shift in increasing number of AOM cases occurred in young children caused by 35B, that was associated with changes in genetic composition and antibiotic susceptibility.

## Introduction

*Streptococcus pneumoniae* (*SPN*) is a common bacterial cause of non-bacteremic pneumonia, sinusitis, otitis media, bacteremia, and meningitis (McCoy and Pettigrew, 2003; Bradley et al., 2011; Mufson et al., 2012). Although pneumococcal conjugate vaccines have had a dramatic protective effect against invasive pneumococcal disease (IPD) (Whitney et al., 2006; Pilishvili et al., 2010), non-IPD (Eskola et al., 2001; Black et al., 2002; Esposito and Principi, 2014), and nasopharyngeal (NP) carriage of vaccine serotypes (Kayhty et al., 2006; Whitney et al., 2006; O’Brien. et al., 2007; Pilishvili et al., 2010), strains expressing serotypes not included in the vaccines have emerged as replacements to colonize the NP and cause disease (Cohen et al., 2015; Kaur et al., 2016; Yildirim et al., 2017; Pichichero et al., 2018).

Since 2006, our group has been tracking *SPN* NP carriage and used acute otitis media (AOM) as a sentinel disease in children to monitor prevalence of pneumococcal serotypes (Pichichero and Casey, 2007; Pichichero et al., 2008; Casey et al., 2010; Casey et al., 2013; Kaur et al., 2016; Kaur et al., 2017; Pichichero et al., 2018; Kaur et al., 2020). After implementation of PCV-13 in 2010, we reported that serotype 35B *SPN* became one of the most frequent colonizers in young children (Kaur et al., 2016). However, we rarely detected 35B in middle-ear fluids (MEF) collected by tympanocentesis at the time of AOM prior to 2015. From 2006 through 2016, we found 35B isolates required increased concentrations of multiple β-lactam and fluoroquinolone antibiotics to inhibit growth (Kaur et al., 2020). Our observations in children were not geographically limited. Increased isolation of 35B strains from CDC IPD surveillance was also reported (Chochua et al., 2017; Varghese et al., 2019). During 2015–2019, we observed a striking increase in the detection rates of *SPN* 35B during AOM. Serotype 35B became the most commonly detected serotype among asymptomatically colonized young children and the most common cause of AOM during 2015–2019 (Kaur et al., EJCMID, pending revisions). The shift from NP commensal to AOM pathogen of the serotype 35B led us to perform the comparative analyses described here that included determination of antibiotic susceptibility, molecular characterization, and genetic analysis from whole-genome sequence (WGS) data. The objective was to describe dynamics of circulation of serotype 35B and understand the genetic changes associated with the emergence of a more virulent serotype 35B that was associated with pediatric disease.

## Materials and Methods

### Pediatric Study Population, Sample Collection and Processing, Antibiotic Susceptibility, Multi-Locus Sequence Typing

This study involved subjects who participated in our prospective, longitudinal analysis of NP colonization and pneumococcal infections in young children from June 2006 through June 2019 (Casey et al., 2010; Casey et al., 2013; Kaur et al., 2016; Kaur et al., 2017; Kaur et al., 2020). The focus in this study was pneumococcal serotype 35B for 2006–2019.

Under the study protocol, children were enrolled from community-based pediatric practices, mainly from middle-class, suburban communities in the Rochester, New York area (USA) at 6 months of age at seven time points (6, 9, 12, 15, 18, 24, and 30–36 months of age). All enrolled children had received the full primary series of PCV7 or PCV13 before the enrollment. During these well-child check-ups, NP washes (instilling and withdrawing ~2 ml of saline in each nostril with bulb syringe) were prospectively collected and cultured for detection of pneumococci. The details of the study design have been previously described (Casey and Pichichero, 2004; Pichichero and Casey, 2007; Casey et al., 2010; Kaur et al., 2011; Casey et al., 2013; Kaur et al., 2016; Kaur et al., 2017; Pichichero et al., 2018; Kaur et al., 2020). Pneumococcal AOM was used as the sentinel disease to distinguish commensals from virulent strains. For precision, clinical AOM infections were microbiologically confirmed by culture of tympanocentesis fluid. Children were treated with antibiotics, most often with amoxicillin/clavulanate unless the child was allergic to penicillin, in which case cefdinir was typically used.

Written informed consent was obtained from parents prior to enrollment in the study, and the IRB of the Rochester Regional Health System approved the study.

Standard microbiology processing and identification techniques were used for isolation of bacteria including *SPN* from NP and MEF samples. Serotypes *of SPN* were determined using pure cultures of *SPN* by Quellung reaction using Latex pools and serotype-specific pneumococcal antisera (Serum Staten Institute, Denmark). Antibiotic susceptibility testing was conducted for 69% of the (173/250) serotype 35B clinical isolates obtained. When an NP and MEF isolate at onset of AOM were both serotype 35B, only the MEF isolate was included in antibiotic susceptibility analyses as described (Kaur et al., 2020). The susceptibility of *SPN* to antibiotics was determined with VITEK-2 AST-GP68 or GP74 susceptibility cards (bioMérieux) in the clinical laboratories of Rochester General Hospital. Benzylpenicillin, amoxicillin, ceftriaxone, cefotaxime, meropenem, ertapenem, ofloxacin, levofloxacin, moxifloxacin, erythromycin, telithromycin, vancomycin, linezolid, tetracycline, chloramphenicol, and trimethoprim-sulfamethoxazole (TMP-SMX) susceptibility was tested by automated modified broth microdilution. *SPN* strain ATCC49619 was used as a control for each batch of testing. Pneumococci were classified as susceptible, intermediate, or resistant based on 2021 CLSI breakpoints, with oral cutoffs used for penicillin (CLSI, 2021).

Multi-locus sequence typing (MLST) was conducted for 62% (154/250) of the serotype 35B isolates using either standard PCR and Sanger sequencing for seven housekeeping genes (n=82 isolates) or by *in silico* analyses based on their WGS assemblies (n=72 isolates). Sequence types (STs) of *SPN* by PCR method were determined as previously reported (Casey et al., 2013; Kaur et al., 2016; Pichichero et al., 2018; Kaur et al., 2020). For extraction from WGS assemblies, “Sequence query” on PubMLST was used (Jolley and Maiden, 2010), and STs associated with 35B were extracted using the goeBURST algorithm from PhyloViZ (https://online.phyloviz.net/index) to estimate the clonality of the bacterial population (Feil et al., 2004; Francisco et al., 2012). Strains that shared greater than five out of seven MLST alleles were classified as belonging to a clonal complex (CC) (Tyrrell, 2011). Due to cost constraints and other technical issues (*SPN* could not be recovered from saved culture stocks), antibiotic susceptibility (69%) and MLST typing (62%) were confined to a subset of available *SPN* 35B isolates, selected from our repository to be representative of the time frame and sample types of this project.

### WGS and WGS-Based Analysis

WGS was mainly performed by Illumina. Subsequent sequencing was conducted using PacBio (Pacific Biosciences) for some isolates to ensure higher contiguity and out of convenience, due to multiplexing requirements of our sequencers. Barcoded Illumina sequencing libraries (insert sizes ~150–600 bp) were produced using Nextera XT kits according to the manufacturer’s protocols, and pooled libraries were sequenced on a NextSeq500 at 2 × 150 nt, targeting 200-fold genomic coverage per library. Barcoded PacBio SMRTbell libraries (insert sizes ~3–20 kb) were produced according to protocol for sequencing on the PacBio Sequel instrument, targeting 100-fold genomic coverage per library. We performed WGS sequencing on a subset of our isolates (**Technical Appendix 1**) to identify potential genetic factors within ST558 associated with the emergence of serotype 35B isolates in pediatric AOM cases and to validate a putative 15A-to-35B capsular serotype switch. This included ST558 isolates from pre-2015 (13 isolates) and post-2015 (36 isolates), ST14687 (one isolate), ST156 (seven isolates), ST10493 (eight isolates), and the novel 35B/ST14683 (one isolate) associated with 15A/ST63 (five isolates) collected for our patient cohort over the same time period to act as a basis for comparison.

### Genome Assemblies and Annotation

All sequence data and assemblies have been deposited at NCBI under PRJNA734910. Illumina reads had technical sequences, such as barcodes and adapters, removed by Trimmomatic (v 0.33) (Bolger et al., 2014). Paired ends that overlapped were merged into single reads using COPE (v 1.1.2) (Liu et al., 2012). Merged and unmerged reads had errors corrected using ErrorCorrectReads.pl from allpathslg. Corrected reads were assembled using Spades (v 3.7.0) (Bankevich et al., 2012). Heavily contaminated assemblies were removed by dropping those that were not *SPN* according to Taxator-tk. This was done before removing bad contigs because, in our experience, small amounts of contamination can be removed by contig filtering, but this is more difficult as the level of contamination increases. Poor quality contigs were removed by removing those in which more than 97% of the contig was a single nucleotide, the total length was less than 256 bases, or the coverage per million reads remaining after Trimmomatic was less than 4. Genomes were removed from further analysis if they had more than 130 contigs remaining.

PacBio reads were assembled using Falcon (smrtlink v 7.0.1) (Chin et al., 2016). Contigs were circularized using Circlator (v 1.5.5) (Hunt et al., 2015), and then the resulting assemblies were polished using Arrow (smrtlink v 7.0.1). Contigs were removed if their normalized coverage was less than 0.06 or their quality score was less than 60. The remaining contigs had errors corrected using the corrected Illumina reads (the reads after running ErrorCorrectReads.pl) and Pilon (v 1.23) (Walker et al., 2014). Assemblies for some strains (PP6, PP9, PP11, PP12, PP13, PP14, PP15, PP16, and S589) were not corrected because they were not sequenced on Illumina. Finally, circular contigs were permuted to start at dnaA and linear contigs (only S602) were split into two pieces such that one piece started with dnaA. For subsequent analyses, PacBio assemblies were used if available; otherwise, Illumina assemblies were used.

Before annotation, the species of each assembly was confirmed using Taxator-tk (v 1.2) (Droge et al., 2015). The genes in each assembly were annotated using Prokka (v 1.13) (Seemann, 2014), and then gene clusters were identified using Roary (downloaded from github on January 22, 2019) (Page et al., 2015). Gene clusters with at most one copy per genome were aligned using Prank (v 0.100802) (Loytynoja, 2014) and then concatenated, adding gaps for genes absent from any alignments. This alignment was used as the input alignment to make a phylogenetic tree with RaxML (v 8.2.4) (Stamatakis, 2006). The tree was midpoint rooted using ETE (v 3.0) (Huerta-Cepas et al., 2016). To search for gene presence/absence differences associated with the time periods of interest, we gave the roary gene clusters and the phylogeny to Scoary (v 1.6.16). Both the gene clusters and the phylogeny were filtered so the only contained strains were 35B and ST558.

To find SNPs between the 35B, ST558 strains, we called variants using Snippy (v 4.4.5) (Seemann, 2015). Isolate PP6 was used as the reference strain. For each query strain, we used Illumina reads; otherwise, we used the PacBio assembly for that strain (strains without Illumina reads are listed above). The list of variants was filtered for significant hits using the filtered phylogeny and Scoary (v 1.6.16). These top hits were mapped to genes using Prokka’s gene annotations and SnpEff (v 4.3) (Cingolani et al., 2012). Finally, the gene clusters from Roary were used to map hits between strains. The genes that were not identified by Prokka were further identified by BLAST search.

### Analysis of Capsule Switch From Serotype 15A to 35B

The best candidate for the parent strain was identified by examining the phylogeny and using BLAST (v 2.2.28) with the sequences flanking the capsule. The candidate for the capsule donor was identified by using BLAST with the sequence of the capsule and the surrounding region. Each strain was then aligned to S558 (the strain with the capsule switch) using Mauve (v 2015-02-13) (Darling et al., 2004). In order to improve the quality of the alignments, contigs were first reordered using Mauve’s contig mover. SNPs and indels were extracted, and sliding windows of each were computed using a window size of 1,000 bp and an increment of 100 bp. Capsular serotypes identified by serotyping methods were further confirmed with PneumoCat (v1.2) (Kapatai et al., 2016).

For pilus-1 operon (Regev-Yochay et al., 2010), genes associated with antibiotic resistance [β-lactam: *pbp1a*, *pbp2x*, *pbp2b* (Li et al., 2017), fluoroquinolones: *gyrA*, *parC* (Brueggemann et al., 2002), macrolides: *ermB*, and tetracycline: TETM (Chochua et al., 2017)] were annotated and extracted with PATRIC. (Brettin et al., 2015; Wattam et al., 2018). PBP typing was classified based on the penicillin binding protein gene type list available at (https://www.cdc.gov/streplab/pneumococcus/mic.html?CDC_AA_refVal=https%3A%2F%2Fwww.cdc.gov%2Fstreplab%2Fmic-tables.html) as of February 2021. For fluoroquinolones, where extracted gene sequences have previously reported point mutations on *gyrA* and *parC* were analyzed (Brueggemann et al., 2002). The presence of the *ermB* gene and TETM were checked using PATRIC.

### Statistical Analysis

The difference in the case (AOM) to colonization ratio (the number of isolates from MEF at the time of AOM/the number of isolates from NP at the time of health) was calculated for each respiratory year (June–May). The ratio between pre-2015 (June 2006–May 2015) and post-2015 (June 2015–May 2019) was analyzed by the Mann-Whitney test. The change in ratio among ST558 was analyzed by Fisher’s exact test. The trend in antibiotic MICs was computed using a linear regression model. Comparison of MIC50 values was conducted using the Mann-Whitney test with a Bonferroni correction.

## Results

### Increasing Prevalence in Children of Pneumococcal Serotype 35B Colonization and AOM

**Figure 1:**
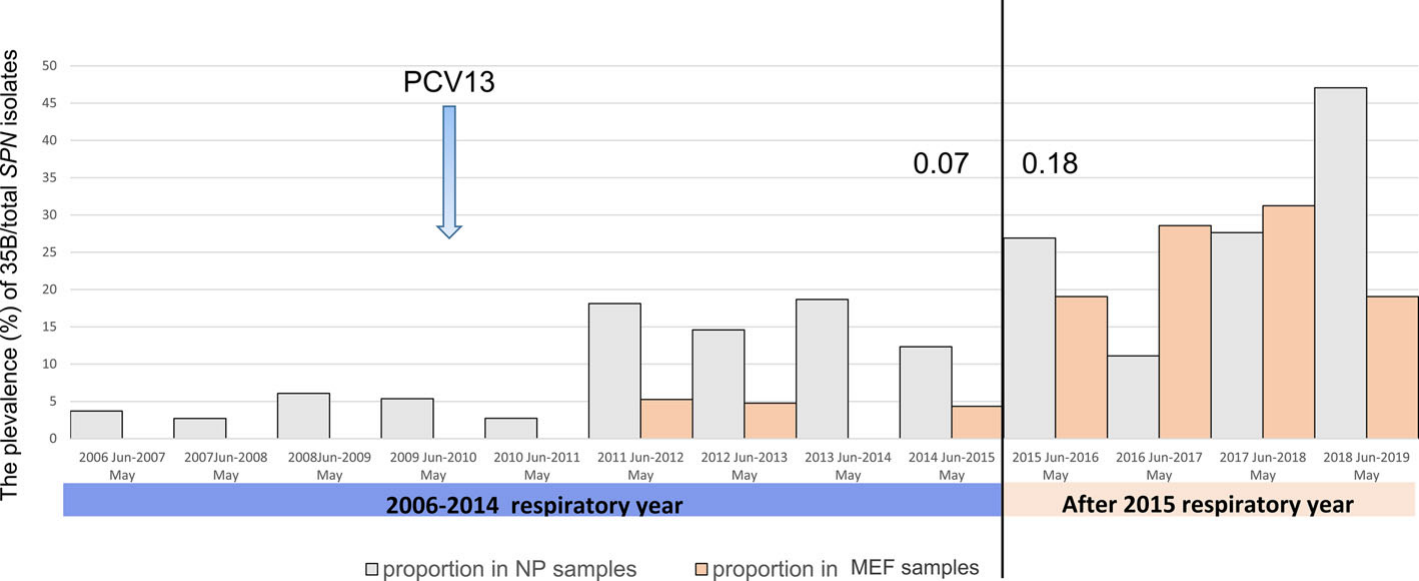
The prevalence of pneumococcal serotype 35B isolation from 2006 to 2019 in the nasopharynx during colonization and AOM and from MEF during AOM disease in 6–36-month-old children. The prevalence (%) of 35B was calculated by dividing the number of 35B isolates with total number of *SPN* isolates. The year is shown by respiratory year (June–May). The case (AOM)-to-colonization ratio (the number of isolates from MEF at the time of AOM/the number of isolates from NP at the time of health) was calculated and is shown for 2014 (0.07) and 2015 (0.18).

We re-examined 250 serotype 35B isolates (consisted of 11.3% of total *SPN* isolates for the study period) that had been collected from 140 children (18.6% of enrolled children) during 2006 to 2019. Of these, 172 isolates (12% of *SPN* isolates) from the NP of healthy subjects, 60 isolates (11.7%) from the NP at the onset of AOM, and 18 isolates (13%) from MEF were collected. Yearly prevalence of 35B isolates as a proportion of all pneumococci is shown in **Figure 1**. Before introduction of PCV13 in 2010, serotype 35B was rarely isolated. After 2011, the relative prevalence of colonizing serotype 35B strains (from healthy NP) significantly increased from 2.7 to 6.1% of all *SPN* (from June 2006 to May 2011) to 12.3–18.8% (from June 2011 to May 2015). Despite colonization prevalence, AOM infections were infrequent. Beginning from June 2015, the prevalence of 35B increased, and it became the most frequently isolated serotype from MEF during AOM bouts. To see the risk of AOM given the frequency of colonization, we calculated the case-to-colonization ratio changes among 35B isolates over time and found AOM cases (MEF isolates during AOM) as a proportion of controls (NP isolates from healthy colonization) significantly increased from 0.08 for the period before June 2015 to 1.68 for the period after May 2015 (p=0.002). Thus, introduction of PCV13 in 2010 was followed by increased prevalence of NP colonization with serotype 35B strains, and an additional shift to higher prevalence and increased AOM disease burden beginning in 2015.

### Serotype 35B Strains Predominantly Belong to a Single Clonal Complex but Capsular Switching Also Occurred

**Figure 2:**
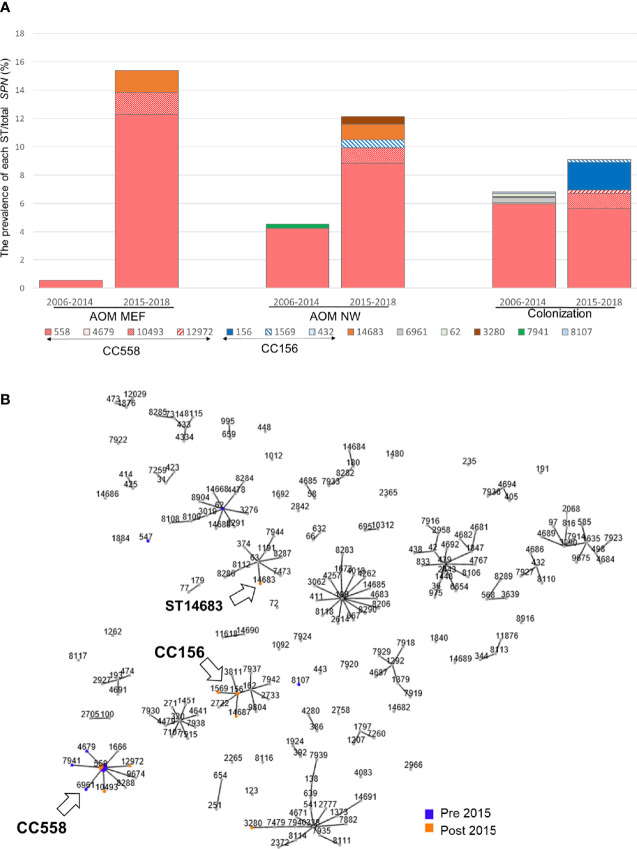
**(A)** Sequence type distribution and prevalence/total *S. pneumoniae* isolates between pre-2015 and post-2015. The prevalence (%) of each ST was calculated by dividing the number isolates with the total number of *SPN* isolates between 2006–2014 and 2015–2018. **(B)** All MLST types and their association with serotype 35B from strains isolated during 2006–2019 in Rochester, NY. Clonal complex that shared greater than five out of seven MLST alleles was linked. Arrow marked 35B/CC558, 35B/CC156, and 35B/ST14683.

Among 163 35B strains tested, 13 distinct sequence types (STs), including two newly identified STs were identified (**Figure 2A**). Most 35B strains belonged to ST558 (89% of pre-2015 and 70% of post-2015 isolates). Although ST558 isolates were detected at comparable levels in healthy children among pre-2015 and post-2015 isolates (6.0 and 5.6%), their isolation among children causing AOM significantly increased post-2015. When the ratio of AOM disease cases to healthy colonization was calculated only among ST558, values for pre-2015 and post-2015 were 0.09 and 2.18, respectively (p=0.0002), underlining the emergent AOM disease burden caused by ST558 *SPN* strains.

To search for evidence of 35B capsular switching, we clustered STs into clonal complexes (CCs) using goeBurst (**Figure 2B**). We found three STs in pre-2015 and two STs observed in post-2015 samples were closely related to ST558 (circled “CC558” consisting of single allele variants from ST558, **Figure 2B**). A more distantly related cluster of three STs separated by a single allele variant including one newly described ST14687 (CC156) was identified; this likely represents a recently described 35B capsule switch between 35B/ST558 and 9V/ST156 serotype strains by Chochua et al. (2017). We also found evidence of a new potential capsular switch. We identified a post-2015 serotype 35B isolate belonging to newly described ST14683, which shares only one allele (ddl) with CC558 and none with those in the “CC156” strains. The ST14683 isolate was detected from two different children. The new ST14683 is a single allelic step from ST63, which was associated with serotype 15A (“CC63”) in our cohort.

### Genomic Comparisons Confirm 35B Association With ST558 and Two Distinct Capsule Switches Into 9V and 15A Genetic Backgrounds

**Figure 3:**
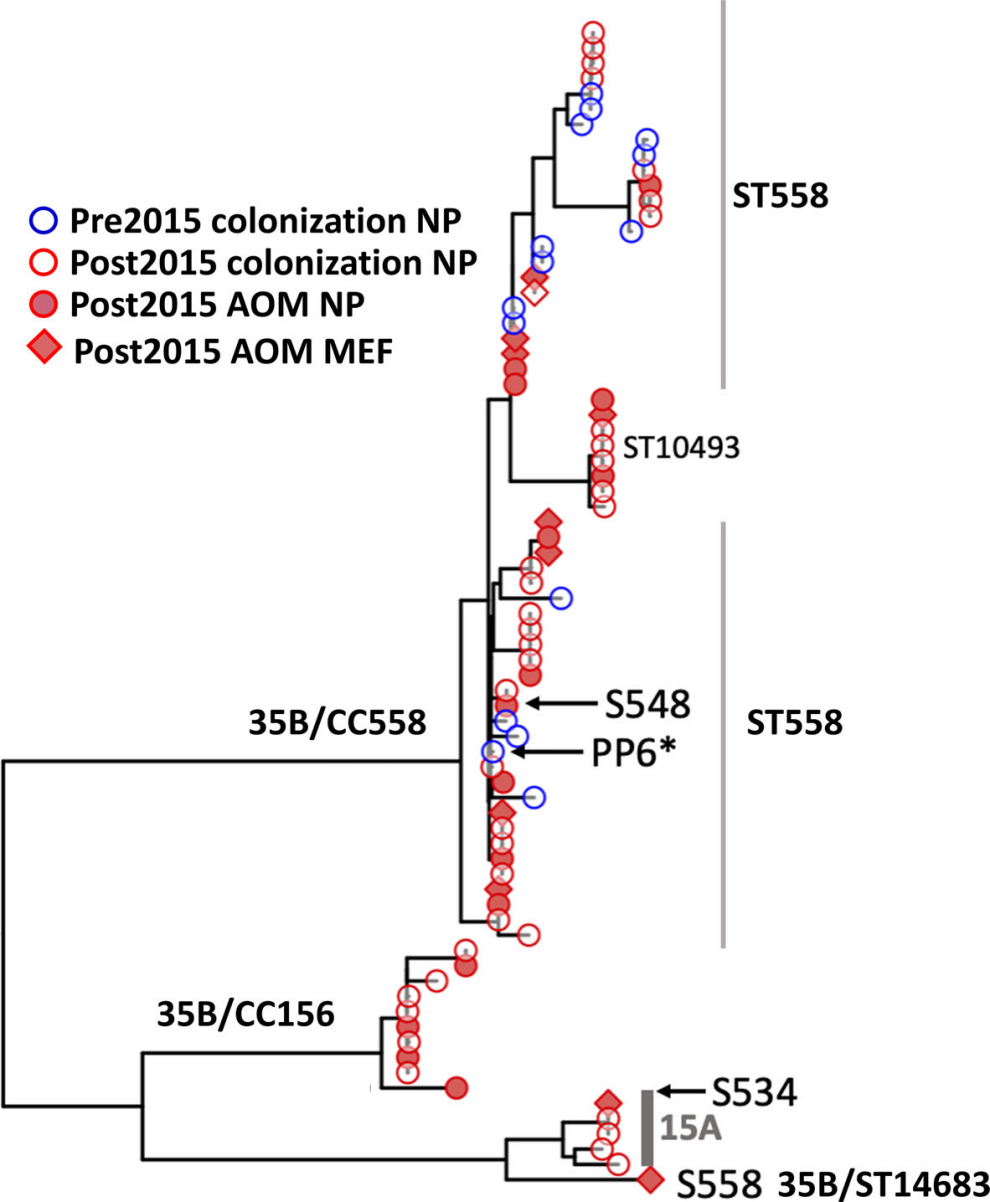
Phylogenetic tree of all 35B isolates based on core protein coding genes from WGS analysis. This phylogeny contains all of the ST558 35B strains sequenced for this study. The strains marked in blue circle were isolated prior to 2015 from colonization and red circle were after 2015 from colonization. Filled red circle represents strains isolated during AOM infections from NP, and filled square represents isolated during AOM from MEF. PP6 used as the reference strain was marked with *. S558 is the 35B/ST14683. The two representative parental strains used for the further analysis to see recombination breakpoints were marked by arrow (S548: 35B/ST558, S534: 15A/ST63).

Genome assemblies were used to produce a phylogenetic tree based on protein-coding genes (**Figure 3**), which found three distinct clades and was consistent with the ST-based results. The largest clade included ST558 and related STs, and strains were isolated from all categories (further discussed below): AOM (MEF and NP) and colonization and pre-2015 and post-2015. The second largest clade contained CC156 isolates that were all collected post-2015, consistent with reports of a recent emergence of this lineage (Chochua et al., 2017). The final branch of serotype 35B consisted of a single ST14683 (S558) strain that was isolated from MEF at an AOM visit that grouped closely with serotype 15A isolates from ST63.

**Figure 4:**
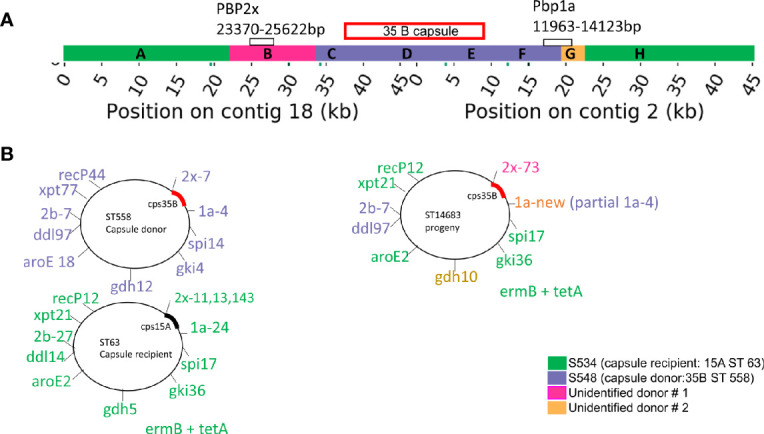
**(A)** Diagrammatic representation of capsule region and adjacent regions from donor, recipient in progeny strain. **(B)** Combination of seven housekeeping genes and PBP genes. The diagram in **(B)** represents our interpretation of the source of each of these regions. The region that is homologous to the capsule donor to the recipient is purple and corresponds to regions C–F in **Supplementary Figure 1**. The regions that are homologous to the recipient strain are orange and correspond with regions A and H in **Supplementary Figure 1**. Regions B (pink) and G (yellow) have limited homology to both the donor and recipient, which probably means that they came from one or more unidentified donors.

To confirm this novel capsule switch and reconstruct the recombination breakpoints, we produced and aligned nearly finished genomes for the 35B/ST14683 capsule switch strain S558 and two representatives of the parental strain backgrounds (for the donor, 35B/ST558 isolate S548, and for the recipient, 15A/ST63 isolate S534). Comparison of nucleotide divergence across the capsule locus and its flanking sequences shows near-perfect identity between the capsule locus and its immediate flanks with the 35B donor representative, whereas the more distant flanks are nearly identical to the 15A recipient representative (**Figure 4A** and **Supplementary Figure 1**). The precise breakpoints were obscured by high divergence from both parents at intermediate distances from the flanks, including the presence of indels, suggesting the involvement of a third donor or more complex history of divergence within 15A strains. Consistent with past recombination at other loci from 35B/ST558 and other strains into ST14683, most MLST alleles in ST14683 were shared with 15A/ST63 strains, but also included two alleles shared with ST558 and three novel alleles, including a novel potential recombinant allele of *PBP 1a* (**Figure 4B**). This ST isolate was negative for *rrgC* (PI-type 1) that is a common character of 15A and positive for ermB and tetM that were the same profiles as 15A ST63 detected in our cohort.

### Associations of Clinical Provenance With Genomic Variation Within ST558

In order to identify potential *SPN* genetic determinants that could be responsible for the increased disease burden associated with the 35B strains post-2015, we focused on genomic variation within ST558, since most of our sequenced 35B strains belonged to this ST and these had been isolated from across the study period. The ST558 gene phylogeny did not group strains by time period, colonization/AOM status, or NP/MEF isolation during AOM, indicating that emergent 35B disease burden post-2015 is not due to a specific ST558 clone (**Figure 3**). We next sought to identify whether specific genetic variants—either gene presence/absence differences or SNPs—within the ST558 lineage were associated with isolation from disease post-2015. To examine gene presence/absence differences within ST558, 2,072 clusters of orthologous genes were identified, of which 1,804 (87%) were found in >95% of strains. The remaining accessory (or distributed) genes showed no significant associations with time period or health status (**Supplementary Figure 2**). Because the resurgence of *rrgC* (PI-type 1) positive strains were observed among non-vaccine type over time and proposed to be selective virulence advantages (Croney et al., 2013), we specifically checked for *rrgC*. We confirmed that most of ST558 isolates through the entire study period were positive for *rrgC*.

SNP analyses identified 138- post-2015-occurring SNPs that were not detected from strains isolated prior to 2015 that were significantly associated with the post-2015 era (**Table 1**). These SNPs were distributed among 34 different genes. Ten percent of these SNPs were found within the *nrdG* (metal binding) gene; 9% within the *metP* and *metN* genes (members of an ABC transporter); 6% within the *corA* (Mag2+ transporter) gene; 5% within the *priA* gene (DNA replication); and 3% within the enzymatic gene, *ldcB* (LD-carboxypeptidase).

**Table 1:** Genes with mutations that are unique to ST558 from post-2015.

Function	Gene name	Moderate	Modifier	The number of SNP
Transporter	corA		8	8
	metP		4	4
	metN	1	7	8
Metabolism	idnO		1	1
	manX_2		1	1
	adk		1	1
Protein biosynthesis	fmt	1	1	2
Transcriptional regulator	YesS*		4	4
Genetic information processing	yqeH	1	1	2
	ftsL		1	1
	priA	3	7	10
	ruvA		1	1
	uvrA		2	2
	rplB		1	1
	rplE	1	1	2
	rplN		1	1
	rplV		1	1
	rsmH_1		1	1
	Putative NrdI-like protein		1	1
Enzyme	nrdG	1	13	14
	nrdD	1	3	4
	ribD		2	2
	fpgS_1		1	1
	kdgA		1	1
	Y1_Tnp*	2	8	10
	ypdF		1	1
	YesM*		1	1
	LdcB*	4		4
	LPXTG-anchored hyaluronate lyase*	1	6	7
Membrane	MptD family putative ECF transporter S*	3	5	8
	DUF1129 domain-containing protein*	5	10	15
Function unknown	YesL*	1	3	4
	ykfA		9	9
	yqeH		1	1
	hypothetical protein		3	3

*The gene name was identified by further BLAST search.

A total of 49 isolates were analyzed (pre-2015: 13 isolates, post-2015: 36 isolates). PP6 was used as reference. High, moderate, modifier describes the impact of SNP categorized by SnepEff. High: stop_gain, Moderate: missense_variant, Modifier: intragenic and intergenic_variant.

### Trends in Antibiotic Susceptibility Among 35B Strains

**Figure 5:**
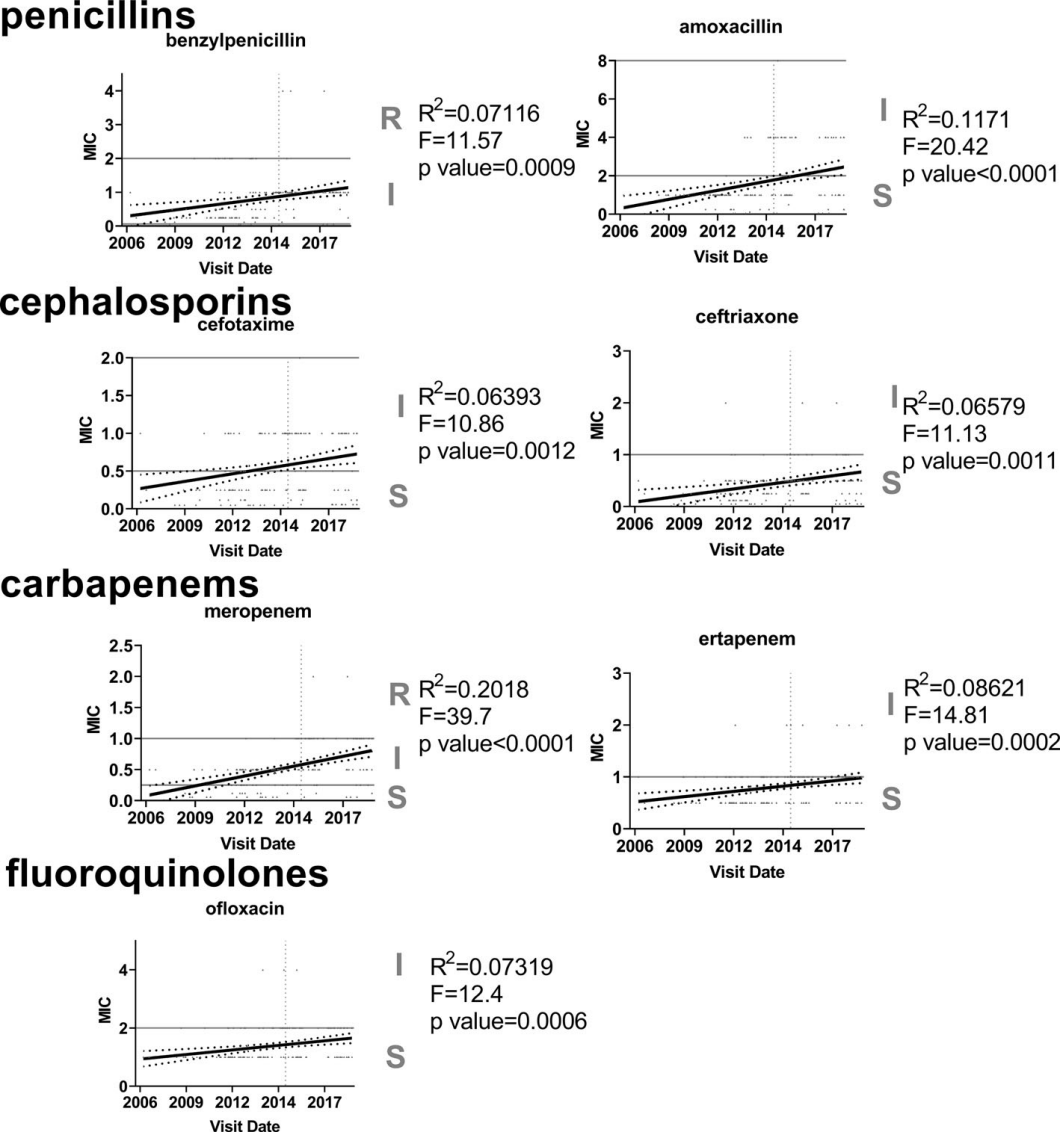
Increasing trend in antibiotic resistance among 35B serotype. Linear regression was used to analyze MIC values (µg/ml) for each antibiotic (n=170). Only seven antibiotics that showed significant increasing trends are shown.

The MICs for 35B isolates significantly increased over time for 7 of 16 antibiotics tested (**Figure 5**). Six of the seven antibiotics were β-lactams, and one was a fluoroquinolone. For amoxicillin, cefotaxime, and meropenem, linear regression analysis showed that the resistance category changed from sensitive to intermediate over the period from 2006 to 2019. The MIC trend for ertapenem and ofloxacin were close to category change (sensitive to intermediate). To identify STs associated with the increase in antibiotic MICs, MIC50s for each ST were also compared (**Table 2**). In general, STs detected after 2015 showed higher MIC50 in six antibiotics (four different β-lactams, ofloxacin, and TMP/SMX). Ofloxacin and penicillin MIC_50_ among ST558 showed significant increases between the two time periods. Although only two isolates were detected as ST14683, it is noteworthy that this ST showed multidrug resistance against 6 β-lactams, ofloxacin, tetracycline, and TMP/SMX.

**Table 2:** MIC50 changes between pre-2015 and post-2015 in each sequence type.

ST	Antimicrobial resistance phenotype, ug/ml																			
	Number of isolates		Amoxacillin		Cefotaxime		Ceftriaxone		Ertapenem		Meropenem		Ofloxacin		Penicillin		Tetracycline		TMP/SMX	
	Susceptibility range (S; I ; R)																	
		Pre	Post	*≤.2; 4; ≥8*		*≤.5; 1; ≥2*		*≤1; 2; ≥4*		*≤1; 2; ≥4*		*≤.25; .5; ≥1*		*≤2; 4; ≥8*		*≤.06; .12-1; ≥2*		*≤2; 4; ≥8*		*≤9.5; 19-38; ≥76*	
			Pre	Post	Pre	Post	Pre	Post	Pre	Post	Pre	Post	Pre	Post	Pre	Post	Pre	Post	Pre	Post
558	73	51	*1*(58)	*1* (24)	*0.25* (60)	*0.25* (25)	*0.25* (60)	*0.5* (25)	*0.5* (59)	1* (25)	*0.5* (60)	*0.5* (24)	*1* (60)	1.5**(24)	*0.25* (60)	1****(20)	*1* (60)	*1* (25)	*10* (60)	*10* (25)
6961	3	ND	*1*(2)	*-*	*0.38* (2)	*-*	*0.25* (2)	*-*	*0.5* (2)	*-*	*0.5* (2)	*-*	*1*(2)	*-*	*0.25*(2)	*-*	*1* (2)	*-*	*10*(2)	*-*
62	2	ND	*1*(1)	*-*	*0.5* (1)	*-*	*0.5* (1)	*-*	*1* (1)	*-*	*0.5* (1)	*-*	*1*(1)	*-*	*1*(1)	*-*	*1* (1)	*-*	*10* (1)	*-*
10493	ND	7	*-*	*2* (5)	*-*	*1* (5)	*-*	*0.5* (5)	*-*	*1* (5)	*-*	*1* (5)	*-*	*2* (5)	*-*	*1* (5)	*-*	*1* (5)	*-*	*10* (5)
156	ND	9	*-*	*4* (8)	*-*	*1* (8)	*-*	*1* (8)	*-*	*1* (8)	*-*	*1* (8)	*-*	*1* (8)	*-*	*1*(8)	*-*	*1* (8)	*-*	*160* (8)
1569	ND	2	*-*	*4* (2)	*-*	*1* (2)	*-*	*1* (2)	*-*	*1* (2)	*-*	*1* (2)	*-*	*1* (2)	*-*	*2* (2)	*-*	*1* (2)	*-*	*120* (2)
14683	ND	2	*-*	*4* (2)	*-*	*1.5* (2)	*-*	*2* (2)	*-*	*2* (2)	*-*	*2* (2)	*-*	*3* (2)	*-*	*4* (2)	*-*	*16* (2)	*-*	*160* (2)
Unknown ST	39	57	*1*(24)	*2* (15)	*0.5* (24)	*1* (15)	*0.38* (24)	*0.5* (15)	*0.5*(24)	*1* (15)	*0.5* (24)	*1* (15)	*1* (24)	2*(15)	*0.75*(22)	*1* (15)	*1* (24)	*1* (15)	*10*(24)	*10* (15)
All 35B	119	131	*1*	2*****	*0.25*	1*****	*0.25*	0.5***	*0.5*	1*****	*0.5*	1*****	*1*	2*****	*0.25*	1****	*1*	*1*	*10*	*10*

Seven antibiotics that showed differences in MIC50 (µg/ml) were analyzed. STs that have more than two isolates are shown. The number of isolates data analyzed for antibiotic susceptibility is shown in brackets. P<0.007 was considered as p<0.05 after the Bonferroni correction. ND, not detected, *<0.05, **<0.007, ***<0.0004, ****=0.0001, *****<0.0001.

### β-Lactams and Fluoroquinolone Resistance Determinants

Since we detected increased MICs in β-lactams and ofloxacin among 35B strains over time, we classified β-lactam resistance determinants (PBP types:*PBP1a, 2b* and *2x*) (Li et al., 2017), and fluoroquinolones resistance determinants (*gyrA/parC* genes) (Brueggemann et al., 2002) in our whole genome sequence data. 35B from pre-2015 only associated with PBP type 4:7:7 except two isolates that had one amino acid mutation on *PBP1a* and *PBP2b*. CC558 (ST558, 10493) from post-2015 was also exclusively associated with PBP type 4:7:7. ST156 and ST3280 were associated with PBP type 4-12-7. Our only sequence from ST14683 had a novel *PBP1a* type that was not found in the list of PBP types provided by the US CDC (**Technical Appendix 1**).

## Discussion

In this study, we report emergence of serotype 35B as a common NP commensal colonizer during 2010 to 2014 in young children and then a dramatic shift to virulence in 2015, evidenced by a significant increase in serotype 35B *SPN* disease in the form of AOM infections. The basic genetic composition of the 35B strains, reflected by MLST analyses, was and remained predominantly ST558 during the switch of the 35B strains from commensals to otopathogens. WGS analysis identified 138 SNPs within 31 different genes that appeared only following the change in virulence in 2015.

Phylogenetic analysis showed that 35B ST558 from pre-2015 and post-2015 were closely related and strains did not segregate by time period. Our data suggest that most 35B isolates before and after emergence of more virulent strains causing disease in young children were from a single clonal complex containing ST558, although two new clonal complexes, including a new switch into a 15A-like genetic background, also were observed. Although the number of detection was small, this capsular switch event between ST558 or ST156 and 15A/ST63 as progeny of ST14683 is noteworthy. Not only the event occurred between two non-PCV13-covered serotypes, but also ST14683 showed multidrug resistance by acquiring tetracycline resistance and accessory gene (TetM) from 15A in addition to high non-susceptibility against β-lactams and TMP/SMX of 35B.

We have found SNPs in 34 genes, including 13 genes with missense variants, were annotated including Transporter genes, metabolism-related genes, enzymes, e.g., *nrdG*, and protein biosynthesis and processing genes. The *nrdG* gene was shown to be upregulated during anaerobic growth of *SPN* R6 strain (Bortoni et al., 2009), suggesting its importance during infection where the concentration of oxygen is low as occurs in the middle-ear space. The *nrdD/nrdG* genes are known to be essential factors in anaerobic growth and induction of biofilm formation in *Escherichia coli* (Garriga et al., 1996; Cendra Mdel et al., 2012). Bacteria change their metabolism based on their surrounding environment in order to acquire essential nutrients to survive and cause disease (Groisman, 1998; Eisenreich et al., 2010). *metP, metN* genes are involved in a methionine ABC transporter (Merlin et al., 2002), and the *corA* gene is involved in a Mg^2+^ transporter (Kehres et al., 1998); both methionine and Mg^2+^ are important for bacterial growth (Shelver et al., 2003; Fontecave et al., 2004; Groisman et al., 2013), and their availability varies on bacteria location (Shelver et al., 2003). Knockout of either of the transporter system showed growth inhibition of *SPN* in various concertation of medium as well as in blood (Neef et al., 2011; Basavanna et al., 2013). ldcB is LD-carboxypeptidases [also known as *dacB* (Cadby and Lovering, 2014)], elucidate the residues essential for peptidoglycan and conformational changes. Deletion of ldcB attenuated D39 *SPN* in murine models of pneumonia and meningitis associated with enhanced uptake by professional phagocytes (Abdullah et al., 2014). Interestingly, 32% of the genes where SNP mutations were found in post-2015 35B strains are known to have functions in genetic information processing such as DNA repair (Ambur et al., 2009). *priA* works in DNA repair by recognizing the DNA replication forks (Nurse et al., 1999; Ambur et al., 2009), and mutated *priA* gene was associated with growth deficiency and more sensitivity to oxidative agents in *Neisseria gonorrhoeae* (Kline and Seifert, 2005). *ruvA* and *uvrA* also function in DNA repair (Iwasaki et al., 1992; Moolenaar et al., 2000). The absence of *ruvA* in *E. coli* leads to a decrease in survival when exposed to fluroquinolone, suggesting the gene functions in phenotypic resistant without becoming genetically resistant (Theodore et al., 2013). The absence of *uvrA* causes hypersensitivity to rifampicin killing (Chao, 1986).

The possible role, association, and importance of the SNPs we identified among recent 35B strains collected from children, and whether they contribute to the virulence of 35B strains, will be the area of future investigation. Sensory-transduction system of *Acinetobacter baumannii* mediates both enhanced virulence by increased protection from serum complement killing and tolerance against antibiotics (Geisinger et al., 2018), consistent with our observation of 35B strains in virulence and antibiotic tolerance.

Among 35B strains, we observed an increase in quantities of six β-lactams and ofloxacin required to inhibit growth. The required MICs for penicillin and ofloxacin significantly increased among 35B strains of ST558 with time, but the increases were not associated with PBP types and fluroquinolone resistance determinants, respectively. Although the increases in MIC for penicillin and ofloxacin did not change the interpretation category of resistance (penicillin: intermediate, ofloxacin: sensitive), which still support the notion that PBP types and fluoroquinolone genetic determinants are the dominant factors that dictate a strain’s resistance to their respective antibiotics (Li et al., 2016). However, our results suggest the existence of additional factors that can modulate antibiotic non-susceptibility at a marginal level. Sequence variations outside the PBPs that influence β-lactam MICs have been reported previously (Sauerbier et al., 2012; Chewapreecha et al., 2014). A recent study has shown that a part of the penicillin-binding protein (*pbp1b*), which does not change resistance to penicillin, but prolongs killing time, increases the risk for pneumococcal meningitis (Li et al., 2019).

The cause of the shift in antibiotic susceptibility to ofloxacin is of interest. Fluoroquinolones are not used in our pediatric population. Together with the unclear reason why we have observed the delayed emergence of 35B among our pediatric population in 2015, we note that in 2014, the CDC recommended use of PCV13 in adults in the USA (Tomczyk et al., 2014), and AOM incidence increase among 35B strains began a year later among our child population, suggesting adult to child transmission of strains. These observations suggest that further study of adult PCV vaccination and adult-to-child transmission of *SPN* may be warranted.

This study had some limitations. Our study was geographically limited. However, 35B emergence has been reported by the CDC as occurring nationwide in the US for both non-invasive and invasive diseases (Chochua et al., 2017). Studies from other groups in France, Israel, and USA have also reported emergence of 35B SPN strains (Cohen et al., 2015; Chochua et al., 2017; Yildirim et al., 2017). We did not sequence all 35B strains, so minority ST populations may have been missed. The causality of genetic differences among 35B/ST558 strains in pathogenesis from pre-2015 and post-2015 was not tested.

In conclusion, serotype 35B strains have become the most frequent pneumococcal disease isolate in young children for the sentinel infection AOM, mainly associated with ST558 since 2015. SNPs in 34 genes were associated only with isolates post-2015. Antibiotic MICs for β-lactams and ofloxacin among 35B strains have increased over time. Emergence and dynamic changes in genetic makeup among SPN strains have occurred since introduction of PCVs, and further changes should be anticipated and studied.

## Data Availability Statement

The datasets presented in this study can be found in online repositories. The names of the repository/repositories and accession number(s) can be found below: NCBI, PRJNA734910. The SRA accession numbers can be found in **Technical Appendix**.

## Ethics Statement

The studies involving human participants were reviewed and approved by Rochester Regional Health Institutional Review Board. Written informed consent to participate in this study was provided by the participants’ legal guardian/next of kin.

## Author Contributions

MP and RK contributed to conception and design of the study. NF and RK organized and analyzed the data. NF, RE, JM, and RK contributed to data curation. NF and JM wrote the first draft of the manuscript. RE wrote sections of the manuscript. GE supervised the whole genome sequence data analysis. All authors reviewed contributed to the article and approved the submitted version.

## Funding

This study was funded in part by NIH NIDCD R01-DC008671 and Sanofi: PI, MP; and NIDCD R01-DC 0428: PI, GE.

## Conflict of Interest

The authors declare that the research was conducted in the absence of any commercial or financial relationships that could be construed as a potential conflict of interest.

## Publisher’s Note

All claims expressed in this article are solely those of the authors and do not necessarily represent those of their affiliated organizations, or those of the publisher, the editors and the reviewers. Any product that may be evaluated in this article, or claim that may be made by its manufacturer, is not guaranteed or endorsed by the publisher.

## Supplementary Material

Supplementary Figure 1 The line graphs of SNPs and indels in the capsule donor and recipient strains when aligned with Mauve to S558, the strain with the capsule switch. The x-axis shows the coordinate on contig 18 or the coordinate on the first 35 kb of contig 2. The y-axis shows the number of indels (top graphs) or SNPs (bottom graphs) in each 1 kb sliding window. The orange lines represent the capsule recipient, S534, and the purple lines represent the capsule donor, S548. Different segments of interest are separated by dashed green lines on contig 18 at 19,600, 30,000, and 34,229 bp and on contig 2 at 3,890, 12,200, and 15,000. The segments are labeled **(A–H)** on the top graphs. **(D, E)** are the 35B capsule.

Supplementary Figure 2 Gene presence/absence difference in ST558 associations with time periods and health status.

Technical Appendix Clinical information, SRA accession numbers, PBP typing, MLST alleles and Phenotypic antimicrobial susceptibility of each isolate determined with WGS is shown.
