# Supplementary figures and images for: Transition of Serotype 35B Pneumococci From Commensal to Prevalent Virulent Strain in Children
Source: Front Cell Infect Microbiol. 2021 Oct 26;11:744742. doi: 10.3389/fcimb.2021.744742 (PMC8577857; doi:10.3389/fcimb.2021.744742)

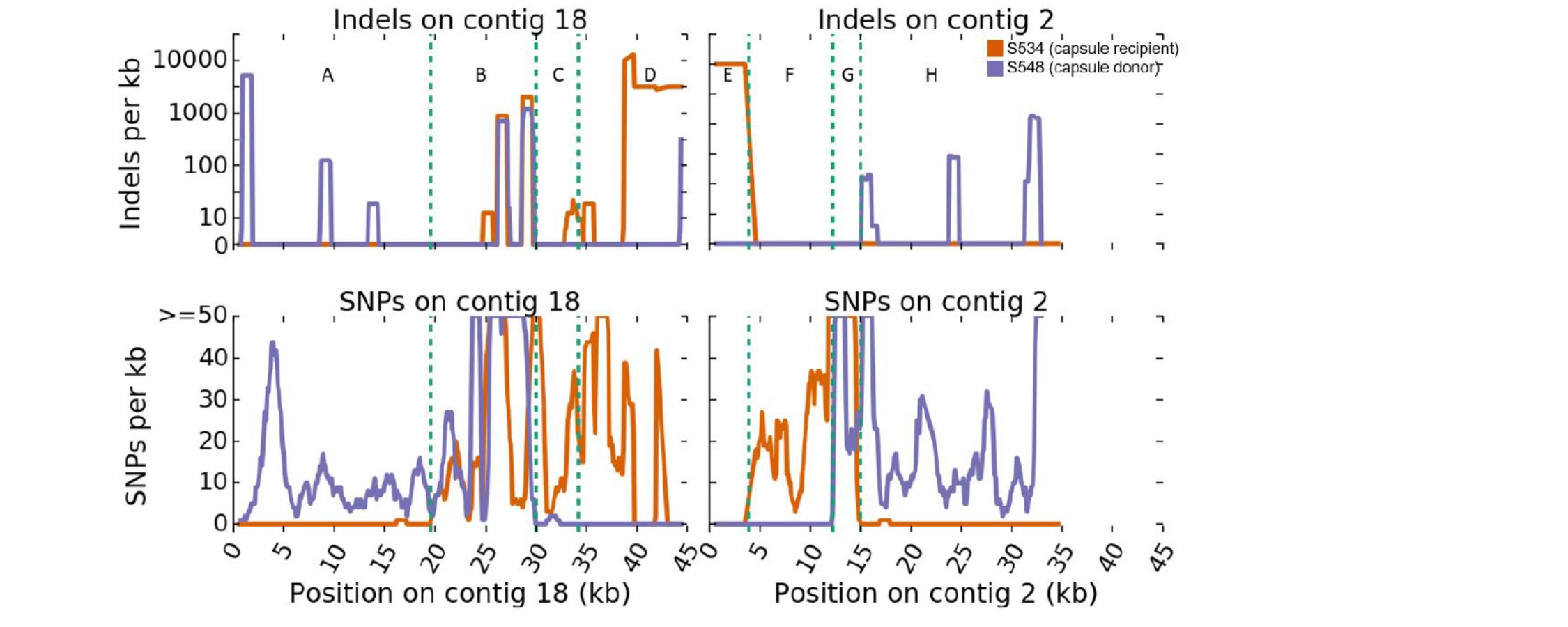

Supplement: Supplementary Figure 1 — The line graphs of SNPs and indels in the capsule donor and recipient strains when aligned with Mauve to S558, the strain with the capsule switch. The x-axis shows the coordinate on contig 18 or the coordinate on the first 35 kb of contig 2. The y-axis shows the number of indels (top graphs) or SNPs (bottom graphs) in each 1 kb sliding window. The orange lines represent the capsule recipient, S534, and the purple lines represent the capsule donor, S548. Different segments of interest are separated by dashed green lines on contig 18 at 19,600, 30,000, and 34,229 bp and on contig 2 at 3,890, 12,200, and 15,000. The segments are labeled (A–H) on the top graphs. (D, E) are the 35B capsule. [file Image_1.jpeg]

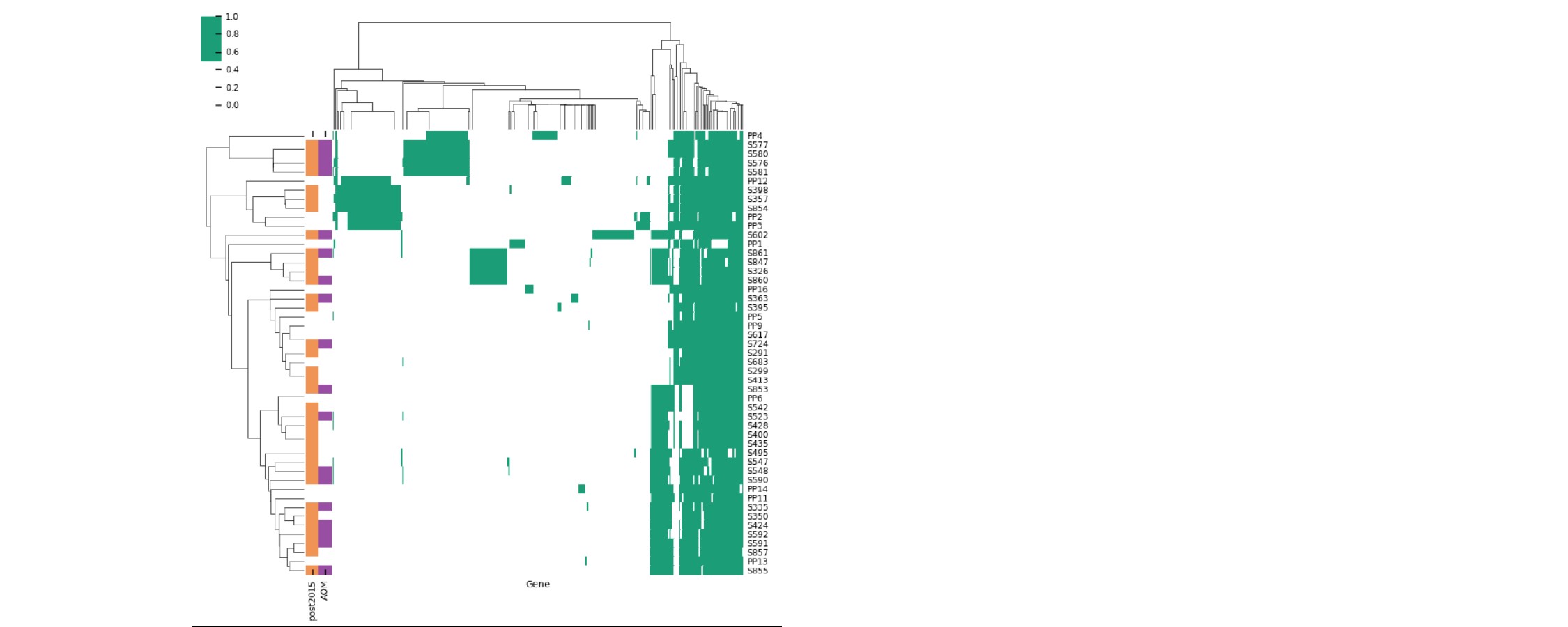

Supplement: Supplementary Figure 2 — Gene presence/absence difference in ST558 associations with time periods and health status. [file Image_2.jpeg]
